# Supplementary material for: Identification of Freezing-Responsive microRNAs and Their Targets in Chinese Jujube by Small RNA and Degradome Sequencing
Source: Int J Mol Sci. 2026 Sep 4;27(17):7912. doi: 10.3390/ijms27177912 (PMC13566890; doi:10.3390/ijms27177912)
Supplement: Supplementary file 1 [file ijms-27-07912-s001.zip › Supplementary Material S2/novel_miR_207.pdf]

Provisional ID : novel_miR_207
Score total : 59.5
Score for star read(s) : 3.9
Score for read counts : 57.7
Score for mfe : 0.1
Score for randfold : -2.2
Score for cons. seed :
Total read count : 125
Mature read count : 56
Loop read count : 62
Star read count : 7

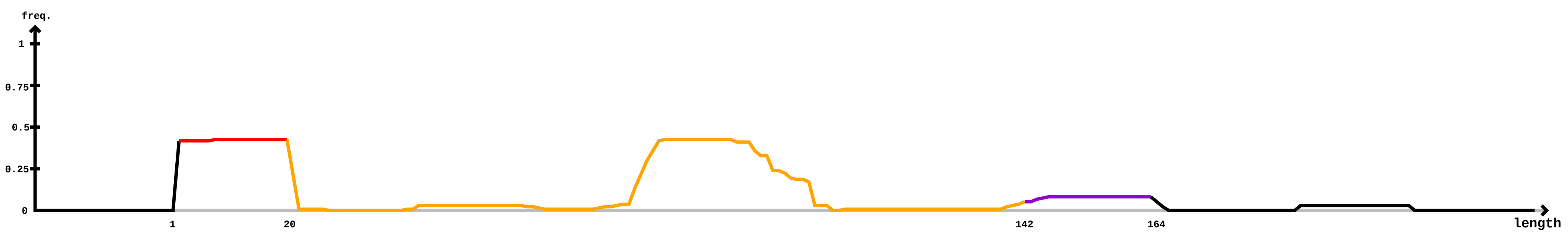

Mature
Star

```
5'- aaaggaaugaaauugaaauuguuuucaauugggucugaauuaaaauugagguuuuauguuucaggauaauguguuugugcaaauaguuugcucaauucaauaccggguugucagagaaaacgcugaugaugcauuuuacgaguugcaaaauccccaagagcagauucaggcuuauguguucgacgguaagucucauacucaaaacuuuuaugggauacuuuuuuuuuuuuuuuuuuuuuuccaaaagucuc -3'   obs
    aaaggaaugaaauugaaauuguuuucaauugggucugaauuaaaauugagguuuuauguuucaggauaauguguuugugcaaauaguuugcucaauucaauaccggguugucagagaaaacgcugaugaugcauuuuacgaguugcaaaauccccaagagcagauucaggcuuauguguucgacgguaagucucauacucaaaacuuuuaugggauacuuuuuuuuuuuuuuuuuuuuuuccaaaagucuc        exp
    ...((((.((((..((((((((..((..(((((((((((....(((.((.(((.(((....(...((((((((...((......((((..((((((......)))))).))))......))....))))))))...).....)))))).)).)))....)))))))))))..))...))))))..((((((((..........))))))))..........)))..)))).)))).........
```

read	reads	mm	sample
uuucaauugggucugaauu	29	0	LCK-2
uuucaauugggucNgaauu	1	1	LCK-1
uuucaauugggucugaauu	26	0	LCK-1
uugggucugaUuuaaaauu	1	1	JCK-2
uAaggauaauguguuugugcaa	1	1	LT-1
aggauaauguguGugugc	1	1	LT-3
aggauaauguguuugugcaaa	1	0	LT-1
aggauaauguguuugugcaaauaguuugc	1	0	JCK-1
ucaauucaauaccggguugucaga	1	0	LT-1
caauucaauaccggguugucagagaaa	1	0	LT-1
aauucaauaccggguugucaga	1	0	LT-2
uucaauaccggguugucagagaaaacgcu	1	0	JCK-3
ucaauaccggguugucagagaa	1	0	LT-2
aauaccggguugucagagaa	5	0	JT-2
aauaccggguugucagagaa	1	0	JT-3
aauaccggguugucagagaaa	2	0	LT-1
aauaccggguugucagagaaa	1	0	LT-2
aauaccggguugucagagaaaac	1	0	LT-1
aauaccggguugucagagaaaacgcug	1	0	LT-1
aauaccggguugucagagaaaacgcugaug	1	0	JCK-2
aauaccggguugucagagaaaacgcugaug	1	0	JCK-3
auaccggguugucagagaaaac	8	0	LCK-3
auaccggguugucagagaaaacgcugaug	3	0	LCK-3
uaccggguugucagagaaaacgcu	3	0	LT-2
uaccggguugucagagaaaacgcugau	1	0	JCK-1
uaccggguugucagagaaaacgcugau	1	0	JCK-2
uaccggguugucagagaaaacgcugaug	1	0	JCK-2
uaccggguugucagagaaaacgcugaug	4	0	LCK-3
uaccggguugucagagaaaacgcugaug	1	0	LT-1
accggguugucagagaaaac	1	0	LT-1
accggguugucagagaaaac	2	0	LT-2
accggguugucagagaaaacgcugaug	1	0	LT-3
accggguugucagagaaaacgcugaug	2	0	LT-1

Mature / Star

```
aaaggaaugaaauugaaauuguuuucaauugggucugaauuaaaauugagguuuuauguuucaggauaauguguuugugcaaauaguuugcucaauucaauaccggguugucagagaaaacgcugaugaugcauuuuacgaguugcaaaauccccaagagcagauucaggcuuauguguucgacgguaagucucauacucaaaacuuuuaugggauacuuuuuuuuuuuuuuuuuuuuccaaaagucuc
.....................................................................................................accggguugucagagaaaacgcugaugaug......................................................................................................................   2   0   JCK-3
......................................................................................................ccggguugucagagaaaacgc..............................................................................................................................   2   0   LT-3
......................................................................................................ccggguugucagagaaaacgcugaug.........................................................................................................................   2   0   LCK-3
......................................................................................................ccggguugucagagaaaacgcugaug.........................................................................................................................   2   0   JCK-1
......................................................................................................ccggguugucagagaaaacgcugaugaug......................................................................................................................   1   0   LT-1
......................................................................................................ccggguugucagagaaaacgcugaugaug......................................................................................................................   1   0   JCK-3
.......................................................................................................cggguugucagagaaaacgcugaug.........................................................................................................................   1   0   LT-3
.....................................................................................................................................uuuuacgaguugcaaaauccccaagagc........................................................................................   1   0   LT-3
................................................................................................................................................................cagauucaggcuuauguguucgacgUu..............................................................   1   1   JCK-3
................................................................................................................................................................cagauucaggcuuauguguucgacgUu..............................................................   1   1   JCK-2
.................................................................................................................................................................agauucaggcuuauguguucgacg................................................................   1   0   JCK-1
.................................................................................................................................................................agauucaggcuuauguguucgacg................................................................   1   0   JCK-2
..................................................................................................................................................................gauucaggcuuauguguucgacg................................................................   1   0   LT-1
...................................................................................................................................................................auucaggcuuauguguucgacg................................................................   1   0   LCK-3
...................................................................................................................................................................auucaggcuuauguguucgacg................................................................   1   0   LT-3
.....................................................................................................................................................................ucaggcuuauguguucgacg................................................................   1   0   JCK-3
.....................................................................................................................................................................ucaggcuuauguguucgacg................................................................   1   0   JCK-1
......................................................................................................................................................................caggcuuauguguucgacg................................................................   1   0   LT-3
.......................................................................................................................................................................aggcuuauguguucgacgUu..............................................................   1   1   LT-3
.................................................................................................................................................................................................................auCggauacuuuuuuuuuuu....................   4   1   LCK-3
```
